# A Case Control Study Evaluating the Relationship between Vitamin K2 Serum Level and Periodontitis

**DOI:** 10.3390/healthcare11222937

**Published:** 2023-11-10

**Authors:** Iwona Olszewska-Czyz, Elena Firkova

**Affiliations:** 1 Department of Periodontology, Prophylaxis and Oral Pathology, Medical Faculty, Jagiellonian University, 31155 Krakow, Poland; 2 Department of Periodontology and Oral Diseases, Faculty of Dental Medicine, Medical University, 4002 Plovdiv, Bulgaria; elena.firkova@mu-plovdiv.bg

**Keywords:** periodontitis, vitamin K2, vitamin K2 serum level

## Abstract

Background and Aim: Vitamin K2 (VK2) is an essential co-factor for bone metabolism. There is still very little data regarding possible VK2 relation to periodontitis. This study aimed to investigate any potential link between VK2 serum level and the severity of periodontitis in comparison to a control group of healthy individuals. The trial was performed on 100 patients among whom 50 were diagnosed with periodontitis. The patients underwent full clinical periodontal and radiological examination. The VK2 serum level was assessed using the ELISA kit (Gla-type osteocalcin EIA Kit, Takara, Kusatsu). Patients with periodontitis had mean serum levels of VK2 significantly lower (0.27 ± 0.06 nmol/L; *p* < 0.001) than the control group (0.43 ± 0.09 nmol/L; *p* < 0.001) regardless of the patient’s age or sex. The VK2 serum level decreased with the severity of periodontitis with the lowest level in stage IV of the disease (0.19 ± 0.01 nmol/L; *p* < 0.001). Also, a significant drop was noticed between the grades of periodontitis. Individuals with localized forms of the disease had significantly lower VK2 levels (0.26 ± 0.006 nmol/L; *p* < 0.001) in comparison to subjects with generalized periodontitis (0.30 ± 0.01 nmol/L; *p* < 0.001). The VK2 serum levels were also associated with most of the clinical parameters such as bleeding on probing (−0.805, 95% CI: −0.894 to −0.654, *p* < 0.001), attachment loss (−0.752, 95% CI: −0.862 to −0.574, *p* < 0.001), and bone loss (−0.656, 95% CI: −0.801 to −0.439, *p* < 0.001). In the present study, the VK2 serum level was correlated to periodontitis, and its severity, complexity, extension, and grade. The range of VK2 was decreasing together with the worsening of all clinical parameters of periodontitis.

## 1. Introduction

### 1.1. Background

Vitamin K naturally occurs as 2 vitamers, K1 (phylloquinone) and K2 (menaquinones, subdivided into multiple isoforms), which have a wide range of biological activities. Vitamin K1 (VK1) plays a role mainly in the production of coagulation proteins, while vitamin K2 (VK2) has a protective role, involved in the functioning of various organs and systems. VK2 promotes bone formation by stimulating osteoblast differentiation, increasing the level and activity of some bone formation markers such as alkaline phosphatase and insulin-like growth factor. It participates in the regulation of bone mineralization and prevention of soft tissue calcification [1]. Further, VK2 might inhibit bone resorption and diminish osteoclastic activity via different strategies, such as reduction in osteoclastogenesis, induction of osteoclast apoptosis, and decrease in RANKL expression [2]. VK2 might additionally inhibit bone resorption induced by bone-resorbing factors such as PGE2 and Il1-α in a dose-dependent manner. As a result of these actions, VK2 shifts the balance towards the formation of bone [3,4].

Scientific evidence suggests that vitamin K2 also has anti-inflammatory activity by inhibition of activation of the nuclear factor kappa B, and thus the decrease in production of proinflammatory cytokines (TNF-α, IL-1) [5]. Recent findings suggest that vitamin K2 has antioxidant properties, which are based on a protective action against oxidative cellular damage, cell death by direct reactive oxygen species uptake, and limitation of free radical intracellular accumulation [6]. VK deficiency is rare in healthy people because it is widespread in food (VK1) and synthesized in the large intestine (VK2); although, it is still not clear if significant amounts of the produced VK2 are absorbed and utilized [7].

According to the World Health Organization (WHO), the recommended intake of VK is 65 µg/day for men and 55 µg/day for women [8]. The EFSA Panel on Dietetic Products, Nutrition, and Allergies sets the adequate intake (both VK1 and VK2) of 70 μg/day for all adults [9]. VK2 supplementation is required in patients taking anticoagulant medications, and people suffering from fat malabsorption or liver diseases. VK2 supplementation is widely used for the treatment of osteopenia and osteoporosis [10].

Significant scientific research is available on the links between VK2 and systemic diseases and conditions, such as osteoporosis, osteoarthritis, rheumatoid arthritis, cardiovascular diseases, diabetes, and even cancer, and cognitive functions [11,12,13,14,15,16]. However, there is not enough data about if, and how it is associated and/or could possibly affect periodontal complex health. Periodontitis is a worldwide spread disease, characterized by inflammation, which is bacterial biofilm-induced, and host-mediated, resulting in progressive destruction of alveolar bone and periodontal attachment loss. Advanced stages of periodontitis and loss of teeth account for a substantial proportion of edentulism, and significant dental care needs [17]. Identifying specific host response-related markers or correlations in health and diseases could be of great importance for comprehensive treatment protocols. In this regard, VK2 status among patients with periodontitis has not been measured yet. Accordingly, a hypothesis of the potential link between VK2 serum level and periodontitis was pre-specified, as well as its possible correlations with the severity of the disease.

### 1.2. Objectives

The study aimed to investigate a possible correlation between VK2 serum level and periodontitis in comparison to a control group of healthy individuals, as well as to analyze a potential link between VK2 volume and the severity, complexity, extension, and progression risk of the disease.

### 1.3. Trial Design

**Figure 1:**
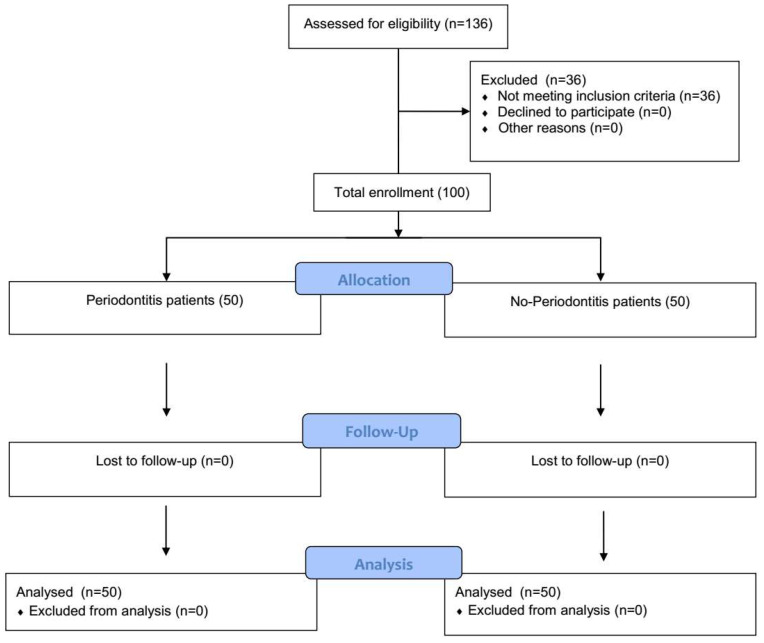
Trial Diagram.

The research was designed as a clinical, prospective, case-control study and performed in one center: the Department of Periodontology, Prophylaxis and Oral Pathology, Jagiellonian University, Cracow, Poland (Figure 1). It was conducted in pursuance of the Helsinki Declaration. Informed consent to participate in the trial was obtained from all the involved individuals. The Ethics Committee approval was granted by Jagiellonian University in Cracow, Poland (No. 1072.6120.313.2018). Enrollment into the project took place during regular appointments at the Department of Periodontology. All the participants continued their periodontal treatment/follow-up plan after participating in the study according to the standard protocol.

## 2. Materials and Methods

### 2.1. Participants

The research was performed on one hundred generally healthy patients among whom fifty were diagnosed with periodontitis while the other half had healthy periodontium. None of the investigated individuals suffered from any diseases nor were they taking any drugs. The additional exclusion criteria were as follows:

- antibiotic therapy within the past 6 months,
- non-steroid anti-inflammatory drugs within the last 3 months,
- corticosteroids within the past 3 months,
- multivitamin supplements within the last 3 months,
- smoking,
- caries,
- epithelial dysplasia,
- inflammatory lesions,
- pregnancy,
- any periodontal treatment,
- any type of pharmacological or radiological therapy within the last 6 months.

The 2017 World Workshop on the Classification of Periodontal and Peri-Implant Disease and Conditions’ Guidelines were applied for the periodontal status assessment, and clinical, and radiological examinations were performed accordingly [18,19].

### 2.2. Data Collection

All the study participants were examined only once during their scheduled appointment at the Department of Periodontology of Jagiellonian University in Krakow, Poland (between October 2020 and October 2021) after giving consent to participate in the trial. The collected data included demographics, medical history, habits (smoking), oral hygiene routine, and periodontal examination.

The periodontal examination was performed by a single clinician and included standard parameters as in the above-mentioned guidelines: Approximal Plaque Index (API), Bleeding on Probing (BoP), Pocket Depth (PD), and Clinical Attachment Level (CAL), as well as Alveolar Bone Level [BL] assessment on radiographs.

Additionally, 4 mL of venous blood for vitamin K2 serum level evaluation was collected from each individual. The samples were centrifuged for 20 min (3000 rpm) straightforwardly and frozen (−20 °C). Vitamin K2 volume was evaluated using the ELISA kit (Gla-type osteocalcin (Gla-OC) EIA Kit, Takara, Kusatsu, Japan), and based on the active and inactive osteocalcin concentration.

### 2.3. Statistical Methods

The IBM SPSS version 27 (2020) software was used for the statistics. The minimum required group size was calculated (46 subjects per group, level of significance 0.05, power 0.9). The Kolmogorov–Smirnov test was applied for normality screening. The serum levels of VK2 between the control and investigated groups were assessed using the Student’s *t*-test, the equality of variances (*p* < 0.05) was checked using the Levene’s test, and the Chi-square test was performed for the categorical variables’ examination. The level of significance was set at alpha = 0.05. The level of significance was adjusted through the Bonferroni test whenever multiple comparisons were carried out.

## 3. Results

### 3.1. Adverse Events and Safety Monitoring

All the study individuals continued their periodontal treatment/follow-up plan after participating in the study according to the standard protocol. No adverse events after periodontal screening or blood sample collection were noted throughout the study.

### 3.2. Study Population

A total of 100 individuals, 50 diagnosed with periodontitis and 50 evaluated as periodontally healthy, were enrolled in the study. The two groups had similar mean age and age range (*p* = 0.924), and almost equal distribution by sex (*p* = 0.841) (Table 1).

In the periodontitis group, 26% of the cases were diagnosed with stage II periodontitis, 10% had initial periodontal destruction (stage I), and only 6% of the cases had clinical and radiographic signs and symptoms of stage IV periodontitis. Of the periodontitis patients, 27% had a moderate disease progression rate (grade B), 12%—grade C, and 11%—grade A. The patients with generalized periodontitis constituted 34%, and 16% were diagnosed with the localized disease, affecting less than 30% of the sites.

### 3.3. VK2 Serum Levels in Investigated Groups (Periodontitis vs. Healthy Controls)

Individuals suffering from periodontitis had VK2 serum levels significantly lower than the healthy individuals (*p* < 0.001). The tendency was consistent irrespective of the subjects’ sex, and age group: the male patients versus the male controls (*p* < 0.001); the female patients versus the female controls (*p* < 0.001); the patients under 50 years of age versus the healthy controls of the same age group (*p* <0.001); and the patients aged over 50 versus the healthy controls of the same age group (*p* < 0.001) (Table 2).

### 3.4. VK2 Serum Levels vs. Periodontitis Stages

**Figure 2:**
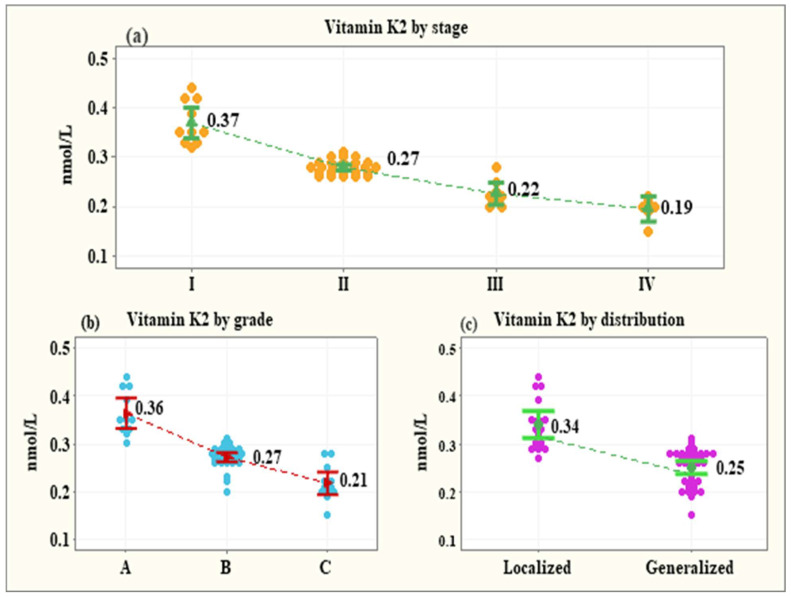
Vitamin K2 levels by the stage of periodontitis (**a**), the grade of periodontitis (**b**), and the distribution of periodontitis (**c**).

The serum levels of vitamin K2 were negatively associated with the stage of periodontitis, its grade, and distribution. The levels of vitamin K2 decreased consistently as the severity of the disease increased (stage I—0.37 ± 0.007 nmol/L; stage II −0.27 ± 0.004 nmol/L; stage III—0.22 ± 0.008 nmol/L; stage IV—0.19 ± 0.01 nmol/L). The differences up to stage III were significant (*p* < 0.001 for all comparisons) except for the difference between stage III and stage IV, *p* = 0.125 (Figure 2a). The grade of periodontitis, excluding the effect of stage and distribution, was negatively associated with a steady decline in vitamin K2 levels. The means of vitamin K2 levels showed a significant reduction from grade A to grade C (grade A: 0.36 ± 0.009 nmol/L; grade B: 0.27 ± 0.006 nmol/L; grade C: 0.21 ± 0.009), *p* < 0.001 (Figure 2b). After removing the influence of the covariates, the mean serum level of vitamin K2 in the patients with generalized periodontitis (0.26 ± 0.006 nmol/L) was shown as significantly lower than the mean serum level of the patients with localized disease (0.30 ± 0.01 nmol/L), *p* = 0.039 (Figure 2c).

### 3.5. VK2 Serum Levels vs. Clinical Parameters of Periodontitis

**Figure 3:**
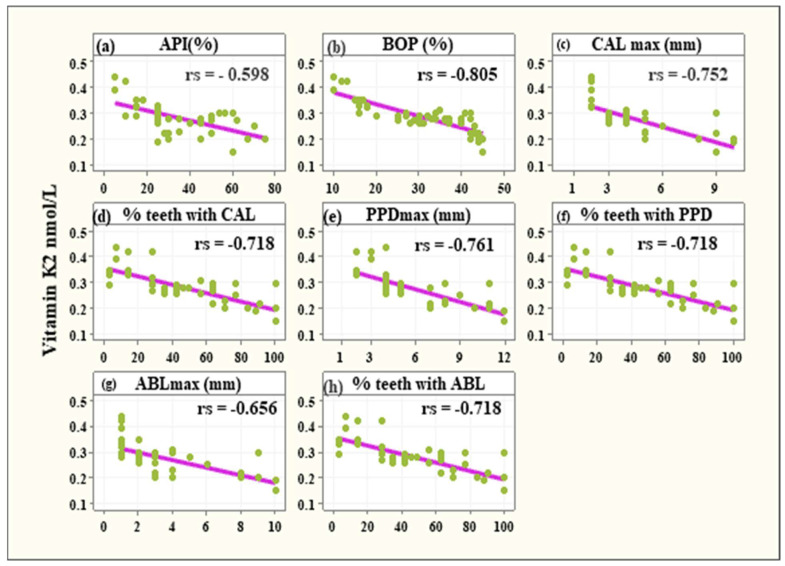
Significant negative associations between vitamin K2 levels and API% (**a**), BOP% (**b**), CALmax (**c**), % teeth with CAL (**d**), PPDmax (**e**), % teeth with PPD (**f**), ABLmax (**g**), and % teeth with ABL (**h**).

In the investigated group (periodontitis) negative correlations were observed between VK2 serum level and:

- Approximal Plaque Index (−0.598, 95% CI: −0.62 to −0.3635, *p* < 0.001),
- Bleeding on Probing (−0.805, 95% CI: −0.894 to −0.654, *p* < 0.001),
- the maximum Clinical Attachment Loss (−0.752, 95% CI: −0.862 to −0.574, *p* < 0.001),
- teeth with CAL (−0.718, 95% CI: −0.841 to −0.524, *p* < 0.001),
- the maximum Pocket Depth (−0.761, 95% CI: −0.868 to −0.587, *p* < 0.001),
- teeth with PD deeper than 3mm (−0.718, 95% CI: −0.841 to −0.524, *p* < 0.001),
- the maximum Alveolar Bone Loss (−0.656, 95% CI: −0.801 to −0.439, *p* < 0.001),
- teeth with ABL (−0.718, 95% CI: −0.814 to −0.524, *p* < 0.001) (Figure 3).

## 4. Discussion

Many studies of vitamin K (phylloquinone—K1, and menaquinones—K2) have been performed in the last years to elucidate their specific role in a variety of essential biologic functions. As vitamin K2 has different types of menaquinones (from menaquinone-2—MK-2—to menaquinone-14—MK14), where the number represents the number of chains of isoprenoid, it plays many roles in the human body [20]. MK-4 is the dominant form of vitamin K and it is synthesized by animal tissues directly from the dietary phylloquinon by conversion of menadione (vitamin K3) and can be found in meat, eggs, and dairy products. It can also be produced by human osteoblasts or synthesized by MK-7 conversion, which has been found to be crucial in bone metabolism and thus the majority of studies are focused on this issue [1]. Many researchers recognized vitamin K2 as an important factor for metabolic changes in the bone tissue, and that is why it is used for the treatment of osteopenia, and osteoporosis, and reduction of the risk of skeletal fractures [21]. It is proved that VK2 efficacy in improving bone quality results not only from its pharmacological features but from structural specificity as well [22]. Researchers showed that it affects osteoblast function, bone resorption, and osteoclast function and that vitamin K is essential in the osteoblasts’ proliferation processes and prevents the induction of apoptosis. It is revealed that due to lowering the expression of cytokines e.g., osteoprotegerin (OPG), and preventing the expression of nuclear factor kappa-B ligand’s activator receptor (RANKL) on bone cells, it stimulates the bone tissue formation process and prevents its resorption [1]. The results of our study follow this pattern of conclusion. Negative correlations were observed between vitamin K2 serum level and the maximum Alveolar Bone Loss (ABL) (−0.656, 95% CI: −0.801 to −0.439, *p* < 0.001), as well as the teeth with ABL (−0.718, 95% CI: −0.814 to −0.524, *p* < 0.001). These numbers were parallel to the fact that the serum levels of vitamin K2 were negatively associated with the stage of periodontitis. The levels of vitamin K2 decreased consistently as the severity of the disease increased with the levels as follows: in stage I—0.37 ± 0.007 nmol/L, in stage II −0.27 ± 0.004 nmol/L, in stage III—0.22 ± 0.008 nmol/L and stage IV—0.19 ± 0.01 nmol/L.

There are many studies regarding K2’s influence on bone tissue. It has been documented that low VK2 serum levels may influence skeletal structure due to a higher risk of osteopenia, osteoporosis, and poor total bone turnover. The conclusion of a study conducted by Kalkwarf et al. suggested that VK supplementation had an impact on better bone tissue turnover in adolescent girls [23]. The above-mentioned authors came across a link between VK serum level, bone formation, and resorption markers; however, they did not reveal a connection between the VK status and the bone mass itself. In another study, Macdonald et al. discovered that VK intake was directly correlated to a higher bone mass density, as well as a reduction in bone tissue turnover indicators in post-menopausal women [24]. Booth et al. found an almost identical relationship [25]. In another research protocol, Feskanich et al. discovered that low VK intake was related to a higher hip fracture risk [26]. Additionally, according to other authors, VK, vitamin D, and calcium supplementation increased lumbar spine bone mass density compared to the control group (only vitamin D and calcium supplementation) [27]. Moreover, many experimental studies highlight the beneficial impact of VK2 on bone formation promotion and the inhibition of its resorption. For example, a study by Akiyama et al. concluded that menatetrenone (vitamin K2 homologue) inhibits bone resorption by influencing osteoclast-like cell formation in mouse bone marrow culture in vitro [28]. In another trial by Wu et al., it was revealed that VK2 regulates osteoclastic activity by reducing osteoclastogenesis, and such action is possible through the inhibition of RankL [29]. Additionally, it was proved by Myneni et al. that normal VK2 serum level enables carboxylation of vitamin K dependent (VKD) proteins, such as Periostin and Matrix Gla protein, thus influencing their activity in different organs. The same pathway takes place in the case of osteocalcin, the most important VKD non-collagenic skeletal protein [30], which, when carboxylated, effectively binds calcium to bone hydroxyapatite crystals [31]. The beneficial effects of VK2 in the regulation of bone metabolism have attracted attention to its potential role in maintaining the health of periodontal structures or promoting periodontal tissue regeneration and remodeling. However, as mentioned above, the clinical studies are focused mainly on evaluating the effects of VK2 on bone metabolism in groups of people with osteopenia and osteoporosis or have an experimental design. Konishi et al. investigated the impact of menatetrenone intake on bone resorption in rapidly progressive experimental periodontitis in rats. The investigators concluded that VK2 supplementation may reduce the number of osteoclasts and thus the bone resorption process itself [32]. Notwithstanding, in another study in rats conducted by Aral et al., the authors proved that vitamin D3 and K2 intake, together or separately in addition to the standard periodontal therapy, did not influence gingival IL-1b and IL-10, serum B-ALP, and TRAP-5b levels, or alveolar bone, when comparing to a control group with standard periodontal treatment only [33]. However, it is questionable to compare the two above-mentioned studies due to the difference in study protocols in terms of timing in VK administration, and experimental periodontitis initiation. The outcomes of our study represent a similar pattern of conclusions. Individuals suffering from periodontitis had a serum level of vitamin K2 significantly lower than the healthy individuals (*p* < 0.001) and the tendency was consistent irrespective of the subjects’ sex, and age. Moreover, in the group of patients with periodontitis, the vitamin K2 serum level was negatively correlated with clinical parameters such as Approximal Plaque Index (−0.598, 95% CI: −0.62 to −0.3635, *p* < 0.001), Bleeding on Probing (−0.805, 95% CI: −0.894 to −0.654, *p* < 0.001), the maximum Clinical Attachment Loss (−0.752, 95% CI: −0.862 to −0.574, *p* < 0.001), and the teeth with CAL (−0.718, 95% CI: −0.841 to −0.524, *p* < 0.001).

Periodontitis is a complex condition with a need for a multidisciplinary approach. Recently, stem cells have become promising sources of achieving the regeneration of periodontal tissue. Some studies investigated the VK2 role in the initiation of the osteogenic differentiation of periodontal ligament stem cells (PDLSCs). The study by Cui et al. investigated the impact of VK2 MK-4 on such activity in vitro and examined the eventual signaling pattern. The trial demonstrated that MK-4 might be responsible for the promotion of the osteogenic differentiation of PDLSCs, with the potential activation of the Wnt/β-catenin pathway. This conclusion may serve as a base for the clinical trials investigating the impact of VK2 in periodontal tissue regeneration [34]. Moreover, another interesting investigation outcome in this aspect was achieved by Vieira et al. The authors proved that the periodontal ligament could differentiate and mineralize into the bone tissue [35], which is parallel to the study results by Arceo and Chou. It revealed that PDLSCs have the ability to differentiate into the bone tissue cells (osteoblasts), and thus can form mineralized nodules [36,37]. The researchers showed that alkaline phosphatase levels were routinely higher in PDL cells compared to gingival fibroblasts (GF). This additional property may be important in initiating mineral formation in vitro. Various studies have shown that mineralization occurs in monolayer cultures of osteoblasts in close association with collagen fibrils. There are however some limitations to such studies. Firstly, the PDL cells and GF may express a tissue culture-dependent phenotype, and secondly, the subculturing of the cells may result in dissimilar populations. Also, the differences in cell function may be related to differences in aging [36]. Many studies underline the relationship between periodontitis and malnutrition and thus vitamin and mineral deficiencies. A Western diet based on ultra-processed food seems to increase the risk of periodontitis [38,39]. Cassiano et al. also showed the association between periodontitis and ultra-processed food consumption [40]. There are also many studies concluding the relationship between periodontitis and serum levels of vitamins such as vitamin D [41] or vitamin C [42]. Parallelly investigators try to assess the influence of vitamins and other supplements intake on the outcomes of the periodontitis treatment and/or progression [43,44,45]. Chuai et al. verified the association between vitamin K intake and the progression of periodontal attachment loss in American adults [46], which corresponds to the experimental results by Aral [33]. The investigators have suggested that VK intake was inversely associated with the progression of periodontal attachment loss; however, the investigators noted some study limitations. Moreover, the grade of periodontitis and the distribution of the disease were negatively associated with a steady decline in vitamin K2 levels. The means of vitamin K2 levels showed a significant reduction from grade A to grade C (grade A: 0.36 ± 0.009 nmol/L; grade C: 0.21 ± 0.009). Also, the mean serum level of vitamin K2 in the patients with generalized periodontitis (0.26 ± 0.006 nmol/L) was significantly lower than the mean serum level of the patients with the localized type of the disease.

To our knowledge, this is the first case-control study measuring the serum levels of vitamin K2 in patients with different stages and grades of periodontitis in comparison to healthy controls. We were not able to find scientifically valid clinical research measuring serum levels of VK2 in correlations with the severity and distribution of the disease. In only one study [46] a cross-sectional analysis based on surveys was conducted. The authors compared only the number of teeth with severe periodontal attachment loss (above 5 mm) to the intake of vitamin K and found that vitamin K intake was negatively associated with attachment loss progression, which corresponds to the results of the current study—VK2 serum levels were associated with attachment loss (−0.752, 23 95% CI: −0.862 to −0.574, *p* < 0.001). Our results demonstrate statistically significant reduced serum levels of VK2 in periodontitis patients compared to healthy controls. The progression of periodontitis and the increase in severity correlate with the reduced VK2 level. There is a significant decrease in VK2 with the lowest level in stage IV and grade C of the disease. Other periodontal parameters, such as bleeding on probing, clinical attachment loss, and bone loss were also negatively associated with VK2 levels. According to the statistical analysis, those correlations are significant regardless of the age of the patients.

As we were not able to compare our results with any published data from similar clinical studies, our findings could serve as preliminary results, demonstrating the association between vitamin K2 serum levels and clinical parameters of periodontitis. Considering that according to the recent studies vitamin K2 out of many functions has an anti-inflammatory effect on the immune system by decreasing the production of pro-inflammatory mediators such as TNF-alpha, IL-1a, an IL-1b its role, it seems to be an interesting correlation for further investigations. As the assessment of VK2 serum levels remains a matter of discussion because there is no unique cutoff indicating its normal value and only indirect methods of evaluation are available, this could limit the assessment of our study results as well.

The impact of vitamin K2 on oral and periodontal health is still unclear. In general, the role of nutraceuticals in maintaining periodontal health is still a huge area for future research. Further studies are necessary to investigate the association between periodontitis and vitamin K2 serum levels to determine if the supplementation along with the standard treatment protocols would have a positive impact on the treatment outcomes.

## 5. Conclusions

In the present study, vitamin K2 serum level was correlated to periodontitis, and its severity, complexity, extension, and grade. The range of VK2 was decreasing together with the worsening of all clinical parameters of periodontitis. Reduced serum levels of VK2 in periodontitis patients compared to healthy controls were observed regardless of the patient’s age or sex. Periodontal parameters, such as bleeding on probing, clinical attachment loss, and bone loss were also negatively associated with VK2 levels. Also, a significant drop was noticed in patients with generalized disease.

## Figures and Tables

**Table 1:** Demographic information about the participants in the study.

Variable	Periodontitis n = 50	Healthy Controls n = 50	*p*-Value
**Age**			
Mean ± SD	49.08 ± 10.14	49.28 ± 10.78	
Minimum–Maximum	25–65	25–65	0.924 ^t^
**Sex n (%)**			
○ Male	25 (50%)	24 (48%)	
○ Female	25 (50%)	26 (52%)	0.841 ^χ2^

^t^—independent samples *t*-test; ^χ2^—Chi-square test.

**Table 2:** Mean serum levels of vitamin K2 (nmol/L) in the patients with periodontitis versus the healthy controls.

Groups	n	Mean (SD)	Mean Diff. (95% CI)	*p*-Value
**Overall**				
○ Periodontitis	50	0.27 (0.06)	0.16 (0.12 to 0.18)	<0.001 !
○ Healthy controls	50	0.43 (0.09)		
**Male**				
○ Periodontitis	25	0.27 (0.04)	0.16 (0.11 to 0.20)	<0.001 !
○ Healthy controls	24	0.44 (0.10)		
**Female**				
○ Periodontitis	25	0.27 (0.07)	0.15 (0.10 to 0.19)	<0.001
○ Healthy controls	26	0.42 (0.08)		
**Age < 50 years**				
○ Periodontitis	22	0.28 (0.06)	0.14 (0.10 to 0.18)	<0.001
○ Healthy controls	23	0.42 (0.07)		
**Age > 50 years**				
○ Periodontitis	28	0.27 (0.06)	8.82 (0.11 to 0.20)	<0.001 !
○ Healthy controls	27	0.43 (0.10)		

Vitamin K2 levels were measured in nmol/L; !—*p*-values for equal-variance not assumed in multiple independent-sample *t*-tests with the Bonferroni adjusted level of significance alpha = 0.01.

## Data Availability

The data presented in this study are available on request from the corresponding author. The data are not publicly available due to ethical restrictions.
